# Heterogeneity in the Beta-Cell Population: a Guided Search Into Its Significance in Pancreas and in Implants

**DOI:** 10.1007/s11892-017-0925-9

**Published:** 2017-08-15

**Authors:** Daniel Pipeleers, Ines De Mesmaeker, Thomas Robert, Freya Van Hulle

**Affiliations:** Diabetes Research Center, Brussels Free University–VUB, Laarbeeklaan 103, 1090 Brussels, Belgium

**Keywords:** Pancreatic islet, Diabetes, Insulin release, Islet transplantation, Beta cells

## Abstract

**Purpose of Review:**

Intercellular differences in function have since long been noticed in the pancreatic beta-cell population. Heterogeneity in cellular glucose responsiveness is considered of physiological and pathological relevance. The present review updates evidence for the physiologic significance of beta-cell heterogeneity in the pancreas. It also briefly discusses what this role would imply for beta-cell implants in diabetes.

**Recent Findings:**

Over the past 3 years, functionally different beta cells have been related to mechanisms that may underlie their heterogeneity in the pancreas, such as the stage in their life cycle and the degree of their clustering to islets with varying vascularization. Markers were identified for detecting these subpopulations in tissues.

**Summary:**

The existence of a functional heterogeneity in the pancreatic beta-cell population is further supported. Views on its origin and methods for its analysis in pancreas and implants will help guide the search into its significance in beta-cell biology, pathology, and therapy.

## Introduction

The co-existence of functionally diverse beta-cell subpopulations was described 30 years ago and proposed to provide complementarity in functions [1]. Subsequent studies led to the concept of a functional heterogeneity in the beta-cell population that determines its physiologic role, that is altered in physiological and pathological conditions, and that explains differential impairment of beta-cell survival and function in diabetes [2–4]. Supporting data were referred to in these review papers, and have since then been added by several laboratories (Table 1). Recent work has focused on underlying mechanisms and on tools for their investigation [5, 6•, 7•, 8•, 9•, 10•, 11•, 12•]. The present review places its contribution in the context of previous observations with the intention to update evidence for the physiologic significance of beta-cell heterogeneity in the pancreas. It also raises the question of what this would imply for beta-cell replacement by implants. This analysis will be conducted along beta-cell characteristics that have been considered so far as the basis for functional diversity (Table 1). It will not provide an in-depth critical dissection of each paper but place the finding(s) in a physiologic perspective, combining observations irrespective of their age.

**Table 1:** Types of heterogeneity observed in the pancreatic beta-cell population, with reported functional significance, some in human (hu) cells

Type of Beta-cell heterogeneity	Functional significance	References
1. Topography in pancreas		
Extra-insular, associated to ducts	Putative sites formation beta cells/small aggregates, no evidence for acute insulin release	[14–18]
Islets in periphery of pancreas	Sites with more amyloid (hu)	[19]
Islets in cauda pancreas	Higher functional responses in vitro	[21, 22]
Capillary-rich versus—poor islets	Higher glucose-induced functions—higher susceptibility to anoxia following isolation	[5, 6•, 7•]
Periphery in islet	Higher cellular hormone content also after sustained glucose stimulation	[1, 20]
2. Nuclear DNA content and synthesis		
Polyploid cells	History of adaptation to sustained metabolic needs (hu)	[23–26]
Ki67+ cells	Response to replication stimuli (hu)	[6•, 32–35]
3. Glucose responsiveness	Distinction glucose-responsive and unresponsive cells (hu)	
Redox state	Dose-dependent metabolic activation of cells > heterogeneity in glucose-dependent functions and in protection against oxidative damage	[1, 2, 39, 45, 48, 49, 55]
Protein synthesis	Heterogeneity in protection against apoptosis—in recruitment of cells into proliferation	[37, 52]
Insulin synthesis and release	Dose-dependent recruitment of cells into insulin synthesis and release (hu)	[43–47, 50]
Membrane electrical activity	Rapid insulin release-coupling for synergy	[8•, 9•, 57, 58]
4. Membrane and cytoplasmic markers		
EM-percent immature secretory granules	Degree of activated beta cells for basal hormone release (hu)	[2, 45, 46, 59]
EM-lipid-containing vesicles	Aging beta cells (hu)	[66]
Fltp (flattop)	Functional maturation versus proliferation-competence	[11•]
E-cadherin; PSA-NCAM	Higher degree of aggregation leading to more potent insulin release	[60–62]
CD9 and/or ST8SIA1 > 4 subpopulations	Differences in insulin release (hu)	[10•]
IGF1R	Aging beta cells (hu)	[12•]

In vitro studies on individual cells help identify the existence of intercellular differences. They should be conducted on sufficiently large samples in order to be representative of the total cell population. Functional analysis of isolated cells can be indicative for the physiologic significance of the observations but will require further evaluations in intact tissue, including human. For the beta-cell population, it will be important to assess whether intercellular heterogeneity contributes to the functional beta-cell mass and its adaptation to varying metabolic needs throughout life [13].

Numerous studies have been conducted on isolated islets and islet cells from different species, strains, age, with cellular compositions and experimental conditions that were often not well defined or standardized. Most were undertaken in the assumption that the studied beta cells were homogenous. The few that paid attention to the possible existence of heterogeneity among beta cells, found it present but differences in study conditions make it difficult to combine data and interpretations in a unifying overview. There is so far also only limited direct evidence on its occurrence at the tissue level and on its pathological or physiological significance. These considerations are however not a reason to minimize or discard the possible relevance of the heterogeneity of the beta-cell population as a mechanistic basis in achieving and maintaining insulin’s homeostatic role. They are inherent to translational research that tests concepts in bidirectional interactions between in vitro and in vivo laboratory models and clinical conditions. From this viewpoint, we will list studies that have demonstrated one or another type of heterogeneity in an adult rodent or human pancreas, irrespective of the in vitro or in vivo model in which they have been identified. They have been selected for their possible functional impact, whether demonstrated or suggestive. Several of them might be closely related in which case their origin and relevance can become easier to further investigate. The present overview is intended to facilitate these studies.

## Types of Heterogeneity in the Pancreatic Beta-Cell Population

### Heterogeneity in Topography

The heterogeneous topography of beta cells in the pancreas is since long known. Several histopathologists reported the occurrence of beta cells as single units or small groups of endocrine cells, disseminated in the exocrine parenchyma, in addition to their characteristic presence in the larger islets of Langerhans, where they are juxtaposed to capillaries and other endocrine cells.

The functional significance of “*islet*” *beta cells* has been investigated in numerous studies; most of them on a collagenase-isolated islet subpopulation with diameter > 200 μm; it is uncertain that these data are representative for beta cells in smaller islets and unlikely that they are for beta cells located outside the islets. *Extra-insular beta cells* appear resistant to sulfonylurea-induced degranulation [14]. They have often been noticed in the lining or proximity of pancreatic ducts [15] raising their possible significance in the formation of new beta cells, be it as remnants of a neogenesis process during pancreas development that can be reactivated later in life [16, 17], and/or as sites from where beta-cell replication can induce formation of small aggregates [5, 18] that can fuse with others under influence of their growth and vascularization.

With islets defined by the presence of capillaries, islet heterogeneity in degree of vascularization, was found associated to differences in local oxygen tension and beta-cell functions in vitro and following transplantation [5, 6•]. It is not known whether such heterogeneity is related to the topography of the islets in the pancreas, and whether it also exists in the human pancreas or develops with the age of the islets. It is conceivable that it is related to preferential deposition of amyloid in islets in the periphery of human organs [19].

The heterogeneous topography of beta cells within islets has been correlated with differences in functions. In rats, those located in the periphery appear more resistant to degranulation following in vivo stimulation by glucose or sulfonylurea [20]; this might be related to their attachment to delta cells, known to release the inhibitory peptide somatostatin [1]. There is so far no in vivo evidence for higher secretory responses of islet beta cells that are located near alpha cells, the source of stimulatory glucagon, but in vitro data clearly show such effect [1, 21, 22].

### Heterogeneity in Nuclear DNA Content and Synthesis

Histopathologists also reported an intercellular heterogeneity in the DNA content, demonstrating the presence of diploid, tetraploid, and octaploid beta cells in the human pancreas; this was not the case for other endocrine islet cells [23]. Polyploid beta cells have also been noticed in normal mice, and found to increase in percentage following prolonged hyperglycemia [24]; a subsequent study in human organs also showed higher percentages in long-standing diabetes [25]. The functional significance of polyploidy in beta cells has so far not been studied. Work in other tissues and cells can serve as guide for such investigation [26]. Laboratory models should assess whether these cells reflect an adaptation to increased metabolic demands and whether this has been successful or not. It is conceivable that beta cells activated into DNA synthesis become polyploidic when not proceeding to replication.

The percentage of beta cells in replicative activity is low in adult human pancreases (< 0.5% as judged by Ki67-positivity), but can increase under higher metabolic demand and in an inflammatory environment [27–33]. The values are significantly lower than those at fetal or young age, which has been related to expression of cell cycle inhibitors [34]. A sharp decline following the neonatal period was also observed in rodent organs [35]. Quantification of cell numbers indicated that this age-related decrease in percentage of Ki67-positive cells was not caused by a decrease in the size of the replicating beta-cell subpopulation but by a massive increase in the number of non-replicating cells [14]. This functionally different subpopulation has not yet been phenotyped and defined for the origin of its cells, in particular in terms of recruitment from non-replicating cells. Beta-cell replication is indeed an ongoing process in an aging pancreas, necessary to achieve and maintain the beta-cell mass of adulthood and to adjust it to elevated needs [36]. In vitro studies on young adult rat beta cells have shown that sustained glucose activation can recruit more cells into DNA synthesis and replication, an effect that is amplified by glucocorticoids; this effect is not seen in beta cells isolated from old rats [37, 38•]. It is so far unclear what makes beta cells susceptible to this recruiting effect.

### Heterogeneity in Responsiveness to Glucose

The availability of purified single beta cells allowed us to compare individual cells for differences in functional activities [1]. Furthermore, the absence of other endocrine and non-endocrine cells avoided their interference with test conditions and interpretations. Our studies were mainly conducted on islet beta cells purified from young adult rats (10 weeks).

In flow cytometry, large numbers of beta cells were individually and simultaneously analyzed for their metabolic state using their FAD and NADPH-related autofluorescence intensity as marker [39, 40]. As a group they exhibited distinct differences with other islet cells, but individual values showed intercellular differences. This became most marked after addition of glucose, which had been previously reported to shift FAD and NADPH redox states in islets during its oxidative breakdown [41, 42]. Glucose rapidly altered FAD and NADPH fluorescence intensity in individual beta cells but a minor subpopulation (< 25%) remained unresponsive, even to high glucose (20 mM or 360 mg/dl), although judged viable by vital staining [39]. Subsequent studies confirmed the existence of glucose-unresponsive beta cells when analyzing secretory or biosynthetic activities at the single-cell level [43, 44].

Among glucose-responsive cells, not all were equally sensitive to the glucose stimulatory effects on their metabolic, biosynthetic, and secretory activities. Glucose was found to dose-dependently increase the number of activated cells [2, 43]. This recruitment process was visualized in flow cytometry, in hemolytic plaque assays and in autoradiographs [39, 43, 44]. Autofluorescence-based cell sorting was used to separate, at a selected glucose level, metabolically active and inactive subpopulations, which were then shown to correspond with secretory and synthetically active and inactive subpopulations [45, 46]. The sigmoidal dose-responsive curves that characterize glucose-induced functions in isolated islet preparations were attributed, at least in part, to a dose-dependent recruitment of beta cells into glucose-dependent activities [2, 4]. The intercellular heterogeneity in glucose responsiveness thus determines in vitro measured functions. It was demonstrated in isolated rat and human beta-cell preparations as well as in the intact rat pancreas [2, 43, 45, 47–50]. Its shift following changes in the in vivo conditions supported its physiologic relevance [4, 51].

Intercellular differences in glucose-regulated redox state were not only correlated with differences in glucose-induced secretory and synthetic activities during subsequent short-term incubations. They also appeared associated with longer-term differences in cellular susceptibility to apoptosis [52] and to proliferation stimuli [38•], processes that highly depend on protein synthetic activity with formation of specific proteins. In proteome analysis beta-cell subpopulations with higher metabolic sensitivity to glucose express higher levels of glycolytic but not of mitochondrial enzymes [53]; this was consistent with their higher expression and activity of glucokinase [54]. Cells that are unresponsive to maximal glucose levels might thus exhibit an inability to increase glycolytic fluxes while maintaining responsiveness to mitochondrial fuels such as glyceraldehyde [55, 56]. It is conceivable that cells without, or with low, glucose sensitivity can be activated through gap junctions by neighboring cells some of which may serve as “hubs,” cells that were recently proposed to synchronize beta-cell responses to glucose within an islet [8•]. Cellular communication between coupled cells helps synergize secretory responses of individual cells [1, 9•, 57, 58]. This calcium-dependent amplifying effect on total hormone release should not be interpreted as proof that intercellular heterogeneity has disappeared, being only an artifact of single cells [9•]. It requires metabolically activated cells, which is calcium-independent. Furthermore, heterogeneity in glucose responsiveness of beta cells has also been observed in islets and intact pancreatic tissue.

### Heterogeneity in Membrane and Cytoplasmic Markers

In vitro observations on cells and tissues face questions on their in vivo occurrence and relevance. This is also the case for data reporting beta-cell heterogeneity in isolated cell and islet preparations. Morphologic markers for in vitro distinguished subpopulations should help to address this issue as they can be applied to the intact pancreas, including human. Several have been identified, thus confirming heterogeneity in situ, and some also give insights in its origin.

Islet beta cells that respond to glucose in the low physiologic concentration range exhibit a higher density of pale secretory granules, which are known to contain more proinsulin [2, 45]. The relative size of this subpopulation increases following sustained activation leading to a higher proinsulin over insulin ratio in the released hormone [2, 45, 46, 59]. An elevated ratio in plasma is thus indicative for a higher proportion of activated beta cells, characterized by higher percentages of immature granules. Islet beta cells were also shown to differ in expression of cell adhesion markers, whereby those with higher expression of E-cadherin or PSA-NCAM were found to be more potent in insulin release [60–62]. Analysis of pancreatic sections for differences in cellular immune-cytochemical staining has indicated additional markers for heterogeneity in the beta-cell population. Recent work has related expression of such markers to differences in transcriptome and intracellular signaling [10•, 11•, 63, 64]. The combination of CD9 and ST8SIA1 identified beta cells with differential gene expression and insulin release characteristics. Dickkopf-3 suggested intercellular differences in Wnt/beta catenin signaling [64]. Flattop (Fltp) marked beta cells that underwent Wnt/planar cell polarity to mature in clustered aggregates, differentiating them from proliferation-competent and glucose-unresponsive Fltp-negative cells [11•].

Differences in beta-cell age can explain heterogeneity for some of these markers [65•] but also for other intercellular differences in the beta-cell population [2]. Not all beta cells in the adult pancreas have been simultaneously formed; the majority is generated postnatally during an ongoing process, possibly with phases of increased rates. The timing of their functional maturation and senescence will probably also vary. The pancreatic beta-cell population is thus likely to be composed of cells in different stages of their life cycle, each with its functional characteristics. Identification of corresponding markers will be useful to demonstrate this basis for heterogeneity and investigate its functional relevance. Recent work helps this approach by identifying Fltp as marker for functionally mature beta cells and IGF1R as marker for their senescence and related functional decline [11•, 12•, 65•]. Human beta cells of age may also be recognized by their lipid storage, visible as lipid storing vesicles in electron microscopy and lipid staining in light microscopy [66].

## Implications for Beta-Cell Replacement in Diabetes

Functional heterogeneity in the beta-cell population is considered to provide adaptation to elevated metabolic needs. This can consist in recruiting more beta cells into insulin synthetic and secretory activity and/or into proliferative activity, which, under chronic activation can lead to hyperplasia and/or hypertrophy of the involved subpopulations. The compensation mechanism should then have resulted in a shift in the heterogeneity profile. When failing to avoid hyperglycemia, this metabolic state will by itself alter beta-cell phenotype(s) with consequences for function and survival. A preceding beta-cell destructive process such as in type 1 diabetes will already have eliminated a large part of the population, conceivably during a long disease process that is also heterogeneous in targeted beta cells.

The view that functional heterogeneity in the beta-cell population represents a key mechanism for its physiologic role, thus raises the question whether and how it can be restored in patients with diabetes. When considering beta-cell replacement by pancreas transplantation, it can be assumed that patients receive a functionally diverse beta-cell population with a habitat that is comparable to that in the donor, and hence maintenance of a regulatory and adaptive potential. This is however less evident for implants of human pancreatic islet cells as these have been enzymatically isolated from their pancreatic topography and placed as mixed endocrine and non-endocrine cell aggregates in an ectopic site. Achieving insulin-independence is by itself not sufficient to conclude that the beta-cell replacement intervention has restored a normal functional beta-cell mass. The hyperglycemic clamp test can be used to compare its size with that in non-diabetic controls. It was thus found that insulin-independent recipients of an intraportal islet cell graft exhibited, at posttransplant month 12, a functional beta-cell mass that was in average 25% of that in age-matched controls [67]; this was markedly lower than that in insulin-independent pancreas-kidney recipients (around 65%), and slowly declined with time. While modified procedures may improve short-term outcome of pancreatic islet cell implants in the liver or elsewhere, their clinically most relevant read-out will be long-term, in preventing diabetes complications. Beta-cell biologists will also be interested to learn whether beta cells that have developed, differentiated, and aged according to a program orchestrated for and in the pancreas, have the plasticity to reorganize towards long-term homeostatic role, following isolation and implantation in another microenvironment.

Phenotypic markers that distinguish beta-cell subpopulations in rodent models and in the human pancreas might be useful for the analysis of beta-cell implants in this context. So far, only few have been identified and examined for their functional significance and their extrapolation to human conditions. Studies on implants in patients are also seriously limited by the scarcity of retrieved tissue. The subject can however be addressed in immune-deficient rodents with implants of human beta-cell preparations are that are similar to those injected in patients. This model can provide data on the functional state of beta cells in the implant in comparison to that in the initial graft and in the intact human pancreas. It can indicate whether markers for beta-cell heterogeneity are maintained, altered or have disappeared, and whether this is related to functional outcome. It will also be interesting to compare these observations with those collected in beta-cell implants that have generated from human stem cells. It is indeed conceivable that implants where beta cells are formed and functionally mature can better adapt to the new microenvironment and the metabolic needs than human beta cells isolated from adult pancreases.

## Conclusion

Heterogeneity in the pancreatic beta-cell population is indicated by beta-cell differences in topography, in nuclear DNA content and synthesis, in responsiveness to glucose, in membrane, and cytoplasmic markers. Most of the described intercellular differences are not subject to rapid disappearance, several shown to be present in the tissue and maintained following isolation. Recent work has related subpopulations to particular stages in the life cycle of the beta cells, and to the degree of beta-cell clustering in aggregates of varying vascularization. Additional markers have been identified to help further investigation of the role of heterogeneity in beta-cell biology, pathology, and therapy. This work can also benefit from an overview of all observations that have been collected so far. Exploring origin and fate of intercellular differences in glucose responsiveness is proposed as a unifying goal.
